# Is it drinking poison to quench thirst? Learners’ risk awareness and continuance intention to use GAI in L2 writing

**DOI:** 10.1186/s40359-026-04330-x

**Published:** 2026-03-12

**Authors:** Beibei Kong, Elanttamil Maruthai

**Affiliations:** 1 School of Foreign Studies, China University of Mining and Technology, Xuzhou, China; 2 Faculty of Languages and Linguistics, Universiti Malaya, Kuala Lumpur, Malaysia

**Keywords:** GAI, L2 writing, Risk awareness, User acceptance, Continuance intention

## Abstract

While numerous studies have emphasized the risks and challenges in integrating AI into higher education, relatively few studies have considered students’ awareness of such risks. What is even more lacking is empirical evidence of students’ awareness of the risks specifically related to their learning. This article reports a mixed-method study to survey the level of risk awareness among a sample of 977 Chinese tertiary learners, assessed the relationship between risk awareness and the intention to continue using AI, and uncover the factors that propel their continuance intention. The article first reported the creation and validation of a two-factor questionnaire of risk awareness: risk of overreliance (RO) and risk of losing teacher feedback (RLTF). Structural equation modelling showed that RO negatively predicted attitude toward AI but positively predicted continuance intention. RLTF positively predicted attitude toward AI but did not significantly predict continuance intention. Interviews suggest that students may deliberately ignore the potential risks in pursuit of course grades and in face of heavy academic work. They believed that the risks were under their control. Implications for AI integration are discussed.

**Supplementary Information:**

The online version contains supplementary material available at 10.1186/s40359-026-04330-x.

## Introduction

The efficacy of artificial intelligence (AI) in higher education has attracted ever more scholarly attention after the emergence of ChatGPT and its rivals [7, 15, 45, 49]. There is growing consensus in the scholarly community that AI will transform the traditional mode of learning and teaching, regarding learning objectives [57], instructional support [34], assessment of learning outcomes [4, 15, 35]. AI conversation chatbots can serve as experts and learning partners, responding to questions and offering instant feedback on student performance [18, 20, 40]. The most impressive feature of AI is its power to generate new content in response to customized prompts [47]. The generative feature of AI, along with its interactive feature, has opened numerous possibilities for education as well as considerable challenges. The immediate concern is the ownership of AI-generated content and its implications for academic dishonesty, such as cheating and plagiarism [8]. Teachers find it extremely difficult to determine the authenticity of students’ submitted assignments and true learning gains [12]. What is even more challenging for writing teachers is to develop and assess the development of students’ language skills [33]. On the one hand, students may uncritically rely on AI to develop ideas, write outlines, create and revise sentences, and find and summarize references [41]. On the other, the AI-generated content may impede language learners’ noticing, a crucial step for language acquisition [25]. Students may pay less attention to key aspects of writing such as linguistic forms or structure because AI-generated content far exceeds their actual levels and requires minimum editing. The potential negative impact of AI could be further complicated by excessive reliance on AI-generated content [11, 54, 56]. Students may reduce in-depth metacognitive engagement and make decisions on their intuition [11]. The risks associated with AI have started to attract scholarly attention [56]. However, the existing literature on students’ risk awareness is largely constrained to ethical risks such as privacy, equity, copyright, and plagiarism [31, 46]. Relatively limited research has explicitly focused on the risk of students becoming lazy [56] or on the perceived risk of losing teacher support because many argue that AI feedback serves as a valuable supplement to teacher feedback [48]. There is a need for more research to explore how students’ perceptions of the risks immediately related to learning might influence their intention to continue using AI in their L2 writing learning. To this research end, this article aims to explore students’ awareness of the risks related to learning and how their risk awareness may affect their intention to continue using AI. As AI could have the largest negative impact on second language (L2) writing, this study purposely focuses on risk awareness in the L2 writing context. The article answers three questions:

1. To what extent are L2 learners aware of the risks of AI to learning?
2. To what extent are L2 learners’ risk awareness linked with their willingness to continue using AI in their L2 writing?
3. What factors propel L2 learners to continue using AI in front of its potential risks?

## Literature review

### Functions and challenges of AI technologies in language learning

Technological advances have considerably improved the affordances of AI technologies. AI applications such as ChatGPT and DeepSeek possess four technological advantages: individualization, interactivity, automation, and accessibility [40, 31]. Language learners can access AI applications anytime and interact with AI agents using personalized prompts. The automated process allows for repeated use until their needs are met, leading many to adopt AI as a learning aid. The most prevalent roles of AI in language learning included generating content, implementing assessment feedback, and acting as collaborative partners [37, 44, 51]. AI can enhance educational content, increase student engagement, and personalize learning [19].

The generative capabilities of AI offer significant potential in L2 writing, assisting students with outlines, idea development, essay revision, proofreading, and post-writing reflection [37]. L2 learners using AI tools like ChatGPT employ more strategies in writing, producing higher-quality texts [32]. Interaction with AI is supposed to increase students’ affective engagement and self-regulated learning [30, 32]. The functions of AI have resulted in a generally positive perception of AI among students. For example, Malik et al., [27] surveyed 245 university students from 25 Indonesian universities about their attitudes toward AI writing tools. The study revealed a positive reception, with students acknowledging benefits in grammar checking, plagiarism detection of AI tools, language translation, and essay outlining. Participants found that AI enhanced their writing skills and understanding of academic integrity. Nonetheless, the existing literature has also brought attention to the challenges of AI integration in the educational context [21, 23]. These include privacy and security issues, academic integrity issues such as plagiarism, low technological readiness, response errors and bias, lack of replicability and transparency, and over-reliance risk [21, 37, 40, 50]. The feedback on writing generated by AI still does not fare as compared to human evaluators [36]. These challenges require teachers and learners to develop competencies to understand the technology, its limitations, and the unexpected fragility of these systems [19]. Misuse of AI may affect our cognitive skills and critical thinking skills [9, 17]. Students are confronted with at least two challenges: to maintain their authentic voice when integrating AI and to avoid losing learning experiences and opportunities that might be caused by AI [40]. In fact, recent research suggests that while use of AI can increase the quality of essay writing, it may have no impact on knowledge gain or transfer [11]. On the contrary, overreliance on AI could trigger “metacognitive laziness” [11]. Recent research highlights students’ concerns about the effects on creativity, critical thinking, and ethical writing [27]. ChatGPT may contribute to procrastination, memory loss, and lower academic performance [1]. Although it can enhance academic performance and higher-order thinking, it reduces mental effort and has little effect on self-efficacy [10]. The seemingly contradictory findings (enhanced academic performance vs. reduced mental effort) could be attributed to a lack of distinction between AI output and students’ learning gains. Because of the increased quality of AI output and academic pressure, students tend to prefer fast and optimal solutions and rely on AI to obtain better academic results [53, 55]. Recent research has documented a moderate level of perceived overreliance on AI, which is positively correlated with AI usage frequency [54].

While the risk of overreliance on GAI is critical for language learning, little attention has been paid to L2 learners’ perceptions of the loss of learning opportunities or external support that may be caused by the integration of artificial intelligence. The existing studies have primarily focused on risks related to ethics or legality [16, 31]. For example, in developing an AI literacy scale, Zhong and Liu , [60] discussed the moral and legal responsibilities of AI developers and the fair mechanisms and regulations for AI application. Despite the importance of these ethical issues, they are not directly relevant to the daily L2 learning context. L2 learners mainly use AI to assist their learning, not necessarily producing texts for public use. Unless including AI-generated content in their work submitted for formal assessment, they do not even commit plagiarism. When using AI as an assistant, L2 learners may be more concerned with its potential effect on their learning outcomes and actual learning gains. Against this backdrop, this study investigates the risks related to language learning, develops a measure of risk awareness as component of AI literacy, and uncovers to what extent L2 learners may continue using AI in their learning in face of the potential risks of learning loss [40].

### Willingness to adopt AI

The existing studies on the willingness to adopt AI for learning have relied mainly on technology acceptance models. Ali et al., [3] identified in their meta-analysis 30 empirical studies that measure the relationship of TAM (Technology Acceptance Model) and UTAUT (Unified Theory of Acceptance and Use of Technology) variables regarding AI technology acceptance in the educational setting. Their study showed that TAM constructs persisted to be robust predictors of user acceptance, whereas some UTAUT constructs were strong predictors (e.g., performance expectancy), some were moderate predictors (e.g., effort expectancy and social influence), and some were very weak (e.g., facilicating condition). L2 research related to artificial intelligence also relies on technology acceptance models [5]. For examle, Liu and Ma , [22] applied the TAM model to survey the intention to use ChatGPT in informal digital learning of English among 405 Chinese EFL learners. PU served as a significant predictor of AT. AT had a statistically significant effect on BI. They found that perceived ease of use (PEU) positively impacted perceived usefulness (PU) but did not predict attitude toward use (AT). Zheng et al., [59] applied UTAUT2 to survey 620 Chinese tertiary EFL learners’ intention to use AI. Performance expectancy, effort expectancy, social influence, hedonic motivation, habit, and motivation significantly were found to predict EFL learners’ intentions toward generative AI tools. These studies generally found perceived usefulness (or performance expectancy) to be a robust predictor of behavioral intention, perceived ease of use relatively weak, and facilitating condition to be insignificant [5, 59].

Recent investigations have also considered additional factors influencing AI adoption such as hedonic motivation and the quality of AI output [28, 58]. For example, Abbas et al., [1] found that academic workload and time pressure positively predict ChatGPT adoption. Wang et al., [43] reported that learning motivations and self-efficacy positively influence AI learning intention. However, these studies have primarily focused on factors that motivate students to continue using AI in their language learning. What has been relatively overlooked are the factors that caution students against becoming dependent on AI [46]. One particularly important factor is awareness of the risks associated with AI, broadly defined as the perception of potential negative consequences or uncertainties related to technology use [56]. The risks of AI range from misinformation, privacy concerns, and social isolation to the potential negative impact on mental effort and metacognitive skills (e.g., Abbas et al., [1], Fan, [11], Han, [16], Ng, [29], Younis, [52], Zhong, [60]. This study, situated within the context of L2 writing, focuses exclusively on the risks of becoming overly reliant on AI and the potential loss of teacher support, as these two factors are directly related to students’ intentions to continue using AI in their future L2 writing learning. To date, few studies have explicitly examined the effects of the risks immediately associated with L2 writing on students’ continuance intention to use AI. One exception is the study by Zhang and Pan [56], which investigated the predictive effects of perceived risks associated with AI use on dependency on AI chatbots. In addition to the risk of misinformation, they also considered the risks of students becoming lazy, less creative, and less critical. Their study showed that perceived risks negatively predict dependency on AI chatbots. Nonetheless, research on perceived risks related to AI remains limited in both scope and quantity. Particularly lacking is research on awareness of the risks of losing learning opportunities or teacher support. As recent research has emphasized the potential risk of overreliance on AI [10, 24, 42, 54], it is imperative to explore the extent to which students will continue using AI to assist their learning when they are aware of its potential detrimental effects [56]. This article addresses this research gap by examining how students’ risk awareness—their understanding of the potential risks of GAI related to overreliance and loss of teacher support—affects their intention to continue using GAI in language learning. Since risks are situation-specific, this article focuses on GAI use in the context of L2 writing. This article has two objectives: (1) develop and validate a risk awareness questionnaire specific to L2 writing, and (2) assess the association between risk awareness and GAI continuance intention.

Based on the existing literature [5, 22, 59], we have the following hypotheses:

- H1. Attitude positively predicts Continuance Intention.
- H2. Risk awareness negatively predicts Attitude.
- H3. Risk awareness negatively predicts Continuance Intention.
- H4. Risk awareness indirectly predicts Continuance Intention through Attitude.

**Fig. 1:**
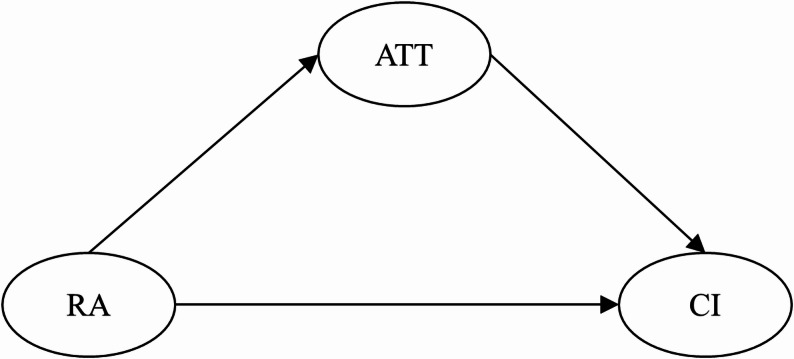
Hypothesized model

The complex relationships are illustrated in Fig. 1.

## Method

### Participants and the context

A purposive and convenience sample of 977 Chinese EFL learners (340 males and 639 females) was recruited for this study. Participants were required to have used AI in their L2 writing for at least one year. The students were drawn from five universities located in Western, Central, and Eastern China. In this sample, 597 (61.0%) participants were English majors, and 379 (39.0%) were non-English majors. Sampling from different regions and majors was intended to increase the representativeness of the sample. The average age was 19.97 years (*SD* = 1.29).

### Instrument

The items for the risk awareness questionnaire were self-developed. There are two sources of the items: (1) the relevant literature on the potential negative impacts of GAI on learning, and (2) proposals from a panel of consultants consisting of applied linguists, language teachers, and students. The panel decided on the concerns most relevant to language learning. In this case, concerns regarding privacy and disinformation were not included because GAI were used for learning purposes, not high-stakes assessment. The initial item pool included over twenty items, but only eleven of them were retained for the present study (see Table 1). The others were removed for irrelevance and redundancy. The self-developed items were included in the Appendix in the Supplementary Materials. The items to measure the attitude toward GAI and continuance intention were adapted from Venkatesh et al., [39]. Attitude is defined as a user’s positive or negative feelings regarding the use of AI, whereas continuance intention to use AI is defined as an individual’s sustained willingness to continue using a specific AI technology after their initial experience with it. The participants were invited to answer the questionnaire on a 5-point Likert scale.

**Table 1:** Factorial structure

	Factor 1	Factor 2
Risk5: GAI reduces my opportunities for independent thinking.	0.853	
Risk7: GAI makes me lazy.	0.817	
Risk6: GAI hurst my learning opportunities.	0.804	
Risk4: GAI makes unable to assess my actual English proficiency.	0.789	
Risk8: GAI hurts my thinking abilities.	0.785	
Risk3: GAI reduces my attention to paragraphing and essay structure.	0.761	
Risk2: GAI reduces my attention to lexis and grammar.	0.705	
Risk1: GAI affects my learning motivation.	0.703	
Risk9: Integrating GAI reduces my chance to receive teacher feedback.		0.863
Risk10: GAI reduces the problems in my writing, which reduces the need for teacher feedback.		0.855
Risk11: GAI makes it more difficult to obtain teacher feedback.		0.672

### Data collection and analysis

Informed consent was obtained from the participants before data collection. The questionnaire was administered from May to June 2025 using *Wenjuanxing*, a Chinese online survey platform similar to *SurveyMonkey*. The participants were invited to complete the questionnaire based on their experience using AI for their L2 writing over the previous month.

The data were closely examined for missing values, outliers, and suspicious patterns. The final sample was 977 valid responses. The dataset was randomly split into two sub-datasets. Dataset 1 was used for exploratory factor analysis to decide on the factorial structure of the questionnaire. Dataset 2 was used for confirmatory factor analysis to assess the reliability and validity of the questionnaire. The full data set was then used to estimate the relationships between risk awareness and user acceptance of GAI technologies in L2 writing. The EFA was performed using SPSS 23.0 and the CFA and structural equation modelling were performed using Mplus 11.0.

A follow-up one-to-one interview was conducted to further explore the relationship between risk awareness and continuance intention. Eight students were invited for the interview. The diversity of the interviewees was maximized regarding their risk awareness and continuance intention. Each interview lasted between thirty and sixty minutes. The interviews were recorded and transcribed for qualitative data analysis.

## Results

### Developing and validating the risk awareness questionnaire exploratory factor analysis

**Fig. 2:**
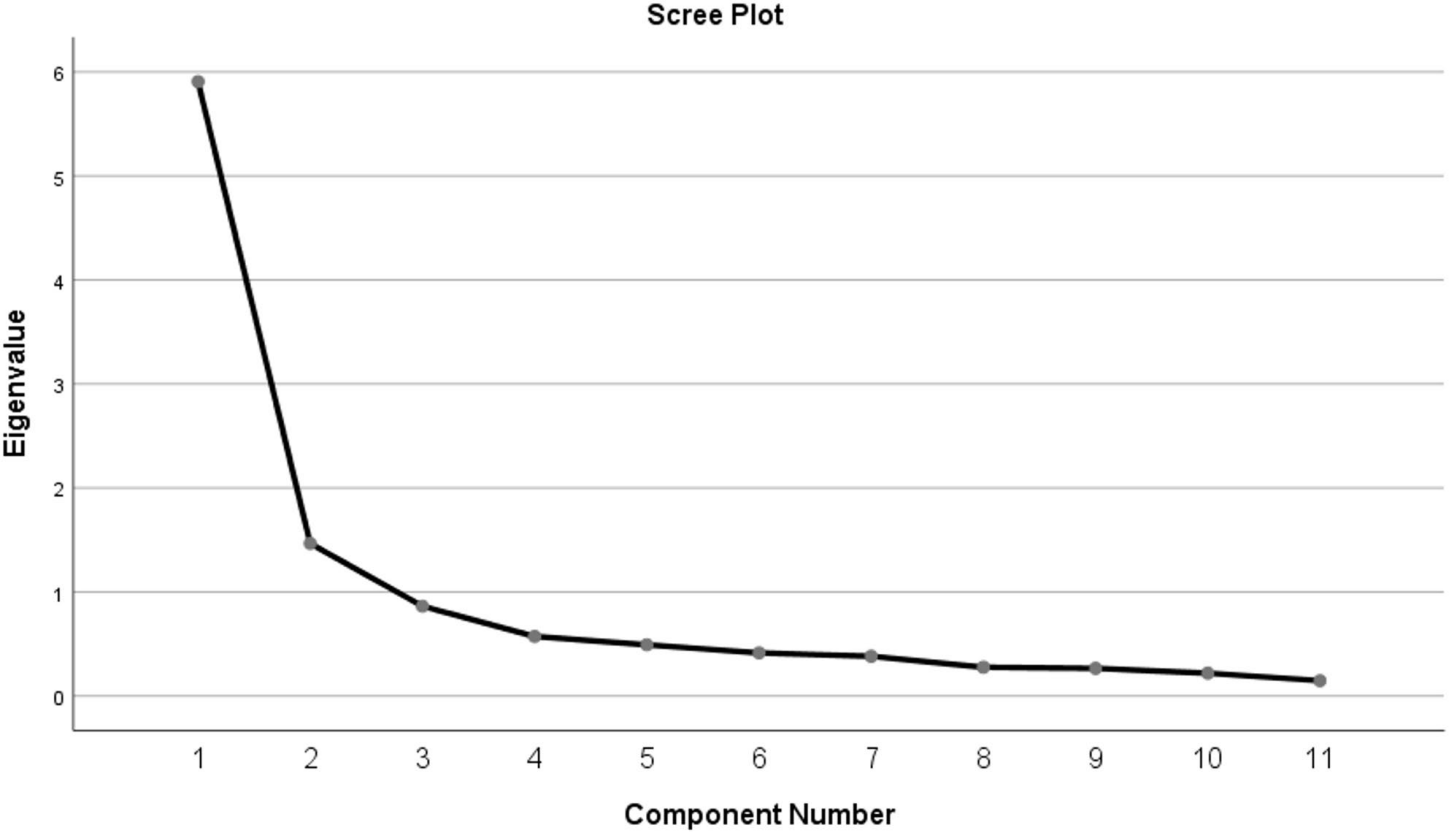
Scree plot

Dataset 1 was processed using principal component analysis (PCA) with varimax rotation. The results of the Kaiser-Meyer-Olkin (KMO) measure of sampling adequacy and Bartlett’s test of sphericity were conducted to evaluate the suitability of the data for EFA. In this case, the KMO value was found to be 0.896. Bartlett’s test of sphericity yielded a chi-square value of 3483.621 with 55 degrees of freedom, which was statistically significant (*p* < .001). Communalities after extraction ranged from 0.494 to 0.767, showing that each item has a reasonable amount of shared variance with the extracted factors. The scree plot (Fig. 2) and rotated component matrix (see Table 1) indicate a two-factor structure.

Two factors were identified, with a cumulative variance explanation rate of 67.025%. This first component has an eigenvalue of 5.907 and explains 53.701% of the total variance, and the second has an eigenvalue of 1.466 and explains 13.324% of the total variance. No additional components meet the eigenvalue threshold. The two factors were renamed as *Risk of Overreliance* (RO) and *Risk of Losing Teacher Feedback* (RLFF). The two sub-scales had an α of 0.922 and 0.776, respectively.

### Confirmatory factor analysis

Dataset 2 was used to confirm the factorial structure. Initial CFA analyses indicate that the measurement model fit was not satisfactory: CFI = 0.944, TLI = 0.928, RMSEA = 0.096, SRMR = 0.035. An inspection of the modification index detected a large MI value of 57.077 because of the overlapping between Risk2 and Risk3. Given that Risk3 also had an MI value over 20 because of its overlapping with Risk6, Risk3 was removed. A second CFA analysis without Risk3 produced improved model fit: CFI = 0.958, TLI = 0.944, RMSEA = 0.087, SRMR = 0.034. While reducing extra items or correlating items can further improve the model fit, we decided not to for two reasons. For one thing, retaining more items can maintain the content validity of the developed measure. For another, correlating items may reduce the replicability of the results.

After removing one item, the whole scale had a reliability of α = 0.918. The two sub-scales displayed good reliability (αs > 0.7 and CRs > 0.7) and convergent validity (AVEs > 0.5) (see Table 2).

**Table 2:** Reliability and validity

Construct	Items	Outer loadings	α	CR	AVE
RO	Risk1	0.699	0.920	0.922	0.630
	Risk2	0.727			
	Risk4	0.787			
	Risk5	0.829			
	Risk6	0.844			
	Risk7	0.825			
	Risk8	0.831			
RLTF	Risk9	0.672	0.773	0.767	0.528
	Risk10	0.636			
	Risk11	0.853			

#### Assessing the relationship between risk awareness and user acceptance

The predictive effects of L2 learners’ risk awareness on their continuance intention to use GAI were estimated using a structural equation modelling. The full dataset was used for this purpose. The data was examined using descriptive statistics. The skewness and kurtosis values indicate that the data did not severely deviate from normal distribution (see Table 3). RO was positively correlated with RLTF. RO was negatively correlated with Attitude but not continuance intention, whereas RLTF was not significantly correlated with either attitude or continuance intention.

**Table 3:** Descriptive statistics and correlations of the latent variables

	M	SD	Skewness	Kurtosis	1	2	3	4
1. RO	3.393	0.845	0.380	0.304				
2. RLTF	3.007	0.832	0.112	0.006	0.563**			
3. ATT	3.721	0.817	0.242	0.141	-0.097*	0.046		
4. CI	3.652	0.727	0.039	0.181	0.040	0.055	0.569**	

*RO* Risk of Overreliance, *RLTF * Risk of Losing Teacher Feedback

### Measurement model assessment

Table 4 shows that the latent constructs in the hypothesized model display satisfactory internal consistency reliability (αs > 0.70 and CRs > 0.70), convergent validity (AVEs > 0.50) and discriminant validity (HTMTs < 0.85).

**Table 4:** Reliability and validity of the constructs

	α	CR	AVE	1	2	3	4
1. RO	0.917	0.917	0.616				
2. RLTF	0.774	0.768	0.526	0.666			
3. ATT	0.859	0.867	0.684	0.118	0.113		
4. CI	0.866	0.867	0.684	0.078	0.072	0.663	

HTMT values

The data was suitable for structural model parameter estimation. The structural model also has acceptable model fit (CFI = 0.944, TLI = 0.931, RMSEA = 0.073, SRMR = 0.047).

**Fig. 3:**
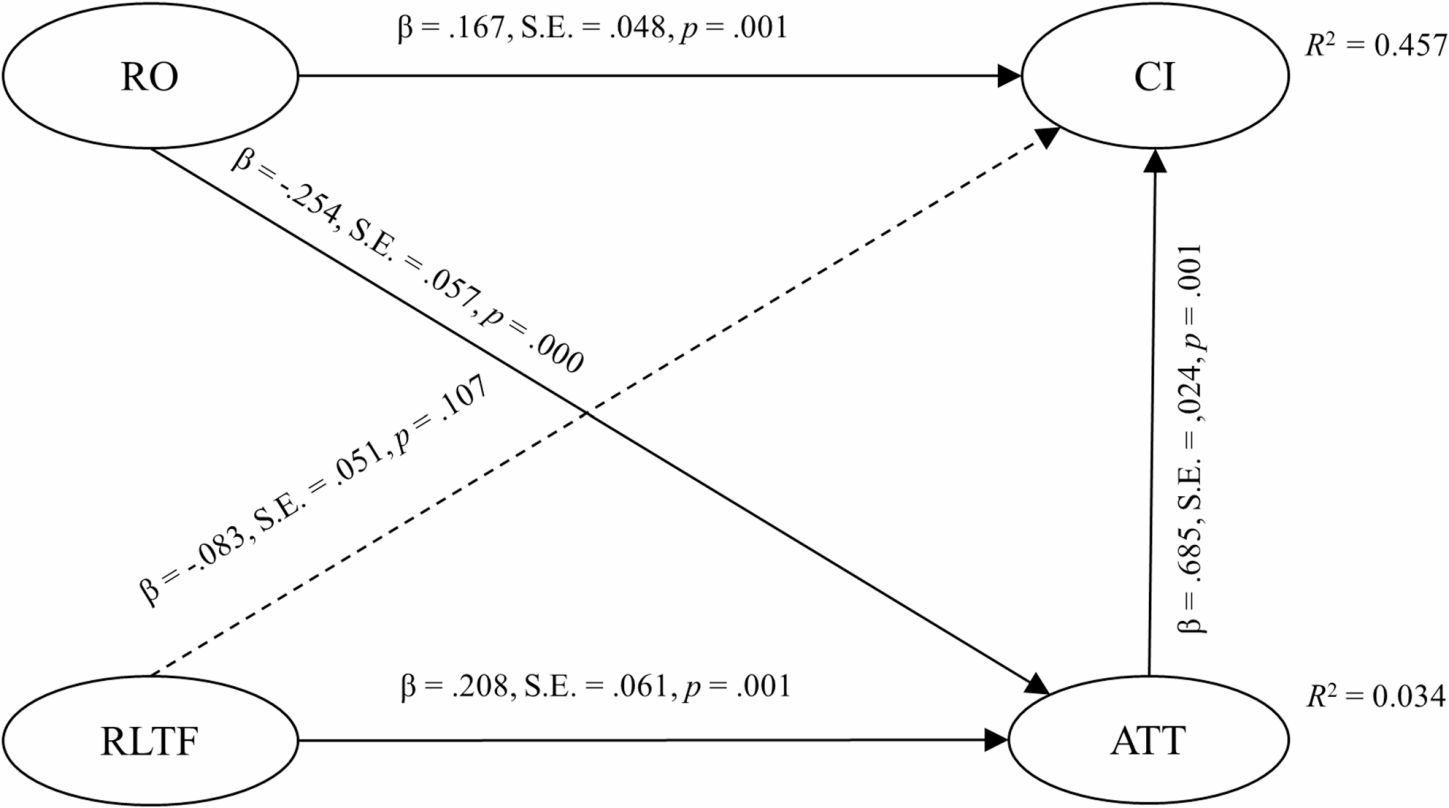
Results of structural equation modelling

SEM analyses indicate that Attitude positively predicted CI (see Fig. 3). H1 was supported. RO negatively predicted Attitude, whereas RLTF positively predicted Attitude. H2 was not supported. RO positively predicted CI, but the effect of RLTF on CI is not significant. H3 was not supported. The hypothesized model explained 3.4% of the variance in attitude, and 45.7% of the variance in continuance intention.

### Mediation analysis

Mediation analysis showed that RO and RLTF indirectly predicted BI through Attitude (see Table 5). H4 was supported. The indirect effect of RO on CI is negative, whereas the indirect effect of RLTF on CI is positive. For the paths from both risk awareness to CI, the indirect and direct effects have opposite signs, constitute what MacKinnon [26] calls a suppression effect. These results reflect the complex relationships between risk awareness, affective attitude, and behavioral intention, a point we will return to when discussing the factors influencing continuance intention in the next section.

**Table 5:** Mediation analysis

	β	S.E.	*p*	95% CI	
RO→ATT→CI	-0.174	0.050	0.001	-0.271	-0.078
RLTF→ATT→CI	0.143	0.054	0.008	0.037	0.249

## Discussion

### The reliability and validity of the risk awareness questionnaire

This article introduced a new two-factor measure of risk awareness related to L2 writing. This measure has expanded the conceptualization of AI ethics from general issues such as accountability, privacy, transparency, and equity to the specific context of language learning (cf. Han and Zhang, [16], Ng, [31], Zhong, [60]. In the context of L2 writing for general purposes, not all ethical issues are relevant to students. The risk related to learning opportunity and support are more robust predictors of students’ continuous use of GAI for L2 learning. The measure displayed satisfactory internal consistency reliability and validity.

### Risk awareness

This study revealed a moderate level of risk awareness. While recent studies have surveyed risk awareness among AI users, few have reported the overall level of risk awareness (e.g., [46, 56]). Consequently, we are unable to compare the level of risk awareness directly and can only discuss perceptions of AI as presented in the existing literature. This result contradicts the prevalent positive perceptions of ChatGPT reported by Tu and Hwang [38], but aligns with the study by Abdelhalim [2], in which students highlighted potential risks such as decreased cognitive abilities. Students acknowledge the risks of overreliance on AI and admit they lack confidence in writing without it:

AI reduces my self-confidence in writing. I feel uncertain when I cannot access AI for English writing. (Xu)

Relying on AI is a stopgap measure akin to drinking poison to quench thirst… Over time, it will undoubtedly lead to a decline in English proficiency (Wang).

This result complements previous studies which observed students’ perceived overreliance on artificial intelligence [53, 54]. While GAI can help minimize the cognitive effort on local issues such as spelling and grammar, this cognitive off-loading does not necessarily contribute to higher order thinking as suggested by prior research (e.g., Zhang and Xu, [54].

### The predictive effects of risk awareness on attitude and continuance intention

The results showed that the risk of overreliance negatively predicted attitude toward AI, while the risk of losing teacher feedback positively predicted attitude. When students were aware of the negative impact of GAI on their learning, they expressed a negative attitude toward integrating GAI into their writing learning.

Sadly, I have developed a rigid writing approach and a stiffened structure characteristic of AI. (Jiang)

When I use AI, I pay less attention to my spelling and grammar. I worry about my English writing when I sit an exam. What if I make many spelling and grammatical errors? (Liu)

Sometimes I feel AI is taking all the pleasure from my learning English. It can write a good essay within seconds. It takes me hours to write a one, but still not good enough. What is the point of learning English? I want AI to do my work. I rest. (Hu)

Clearly, despite the affordances of AI, students were aware of the potential risks in relying on AI to assist their writing. This risk awareness caused a concern and reduced their liking of AI, which had a negative impact on their continuance intention. This speculation is supported by the results from our mediation analysis and then interview excerpts:

I want to stop, but I cannot stop using AI to help me write. But I hold a growing sense of unease. I consistently tell myself “It does not reflect your proficiency level. It is AI.” (Hu).

Sure, I can write better with the assistance of AI. So what? I slide to my actual level when I cannot access AI. I feel unsafe. I think I should stop. (Zhai)

When I used AI to help me write an English essay, I feared that my teacher would ask me “Did you write it yourself?” Even though I carefully edited the content from AI, I still do not feel confident. (Wang)

By contrast, the risk of losing teacher feedback positively predicted attitude toward GAI. This result suggests that students tend to like GAI because they may receive less teacher feedback. One possible explanation is that, compared with teacher feedback, AI-produced feedback causes less emotional cost (e.g., embarrassment) [13]. The students commented on the advantages of AI-generated feedback:

I can seek feedback from AI whenever I want. It never gets annoyed. It is always polite as long as I do not scold it. (Hu)

AI can give me a lot of feedback. It is not like my English teacher. I sometimes fear my teacher because she may criticize my writing. AI also says bad things on my writing, but I do not feel hurt. Perhaps, it is just a robot that I do not care for. (Cai)

When ChatGPT and Kimi became popular, our teacher encouraged us to seek feedback from these artificial intelligence tools before submitting an English essay. It is not bad, at least we can avoid many basic errors, and we no longer receive so much red ink on our essays. Red ink makes me nervous and embarrassed. (Liu)

Unexpectedly, the risk of overreliance positively predicted continuance intention. This finding contradicts Zhang and Pan [56], who reported that perceived risks negatively predicted AI dependency. One possible explanation for this discrepancy lies in the conceptualization of the constructs. Dependency may be perceived by some students as negative and socially undesirable, whereas continuance intention is a neutral construct reflecting behavioral intention. While students may consciously seek to reduce their dependency, they might still continue using AI in their learning due to various factors. The positive effect of the risk of overreliance on continuance intention suggests a maladaptive cycle between subjective perceptions and behavior. In other words, students are aware of the risks associated with overreliance but are unable to regulate their learning behavior effectively. This conflict is further reflected by the indirect negative effect of overreliance risk on continuance intention.

While the risk of losing teacher feedback did not directly predict continuance intention, it indirectly and positively predicted continuance intention via attitude toward AI. This is unexpected because many students believed that the feedback function of GAI may reduce the motivation of their teachers to provide feedback on their writing. In fact, English teachers tend to encourage their students to submit essays to AI systems for feedback. The interviews with students provided nuanced insights into the effects of AI:

Because my teacher thinks AI can provide feedback, he does not comment on my grammatical errors. I feel relieved not to have my essay full of red marks. (Jiang)

My teacher used to pay attention to my spelling and grammatical errors. Guess what? When I avoid such shallow mistakes with AI, she has to comment on my idea and structure. This is what I have always wanted. Now I get it. (Hu)

I can fix my basic errors with AI. So, when I have checked my essay with AI, I can directly ask my teacher about some “bigger” issues, for example, the difference between formal and informal style. (Liu)

From the student perspective, AI does not replace the teacher, but liberate the teacher. The interviews have offered two reasons for the positive indirect effect of AI. First, while GAI was believed by many to be a powerful tool to provide feedback, the feedback quality sometimes cannot fare with teachers [35]. Students still want human feedback and the interaction with their teachers. Second, AI relieved of teachers’ feedback workload. Students feel that they have the grounds to actively seek individualized feedback from their teachers [14]. These results echo previous studies (e.g., Yan et al., [49] which indicated, as compared with receiving teacher feedback, students enacted more low-level revisions in response to computer-generated feedback but showed relatively higher writing confidence and persistence. While the risk of overreliance negatively predicted attitude toward GAI, it positively predicted students’ continuance intention. In other words, despite their negative attitude toward GAI, students still plan to continue using GAI in their L2 writing. This result aligns with Zhang and Xu [54], which observed a positive correlation between AI usage frequency and AI overreliance. The reasons for continuance intention are complex, related to the technological affordances of AI, tendency for cognitive offloading, and school climate. All interviewed participants recognized the affordances of GAI to various extents:

AI can provide me sentence structures that can be used for different essays. The structure of AI-produced essays is clear and coherent. I can learn from AI. (Jiang)

I feel AI is like a teacher that I can call upon any time. It may not be as professional as a real professor, but it can help me complete my homework. (Liu)

When essays produced by AI can score higher than those crafted with arduous efforts, I want to use AI to have a higher score. (Zhai)

The affordances of AI provide a basis for cognitive offloading, i.e., offload thinking to technology to reduce cognitive effort [6]:

I think people are born to be lazy. When AI writes faster than me, I cannot resist the temptation of using AI-generated content, it is better not to write by myself. To be honest, I don’t want to bother. (Cai)

If there is a short-cut, I take the short-cut. For instance, I usually give AI an outline and make it write a draft. Even if AI output is not up to standard, editing is still faster than writing from scratch. (Jiang)

I often let AI write a draft and then take good sentences or words from AI. By this step, I save my time to make sentences. Of course, I will edit the essay before submission. (Fu)

Cognitive offloading is not necessarily maladaptive. It can help learners to re-allocate their efforts and focus on the skills to be developed [11]. The influence of cognitive offloading partly depends on learners’ goal orientations. Some rely on AI to complete assignments and ensure academic performance (Hu), whereas others rely on AI to develop their language skills (Fu):

I used to submit AI-generated essay without any changes. Each time I got a grade of over 90 out of 100. I did not edit the essay because my editing reduced the score. (Hu)

AI can provide advanced phrases. After learning these phrases, I can write better than before. (Fu)

The structure of courses and the number and intensity of assignments have a critical effect on students’ goal orientations:

You have no idea how many assignments we need to do. Who has time to do so much work? We rely on AI as a digital lifeline to complete assigned homework. Before authentic learning, we need to survive the deadlines first. Using AI-generated content is better than not submitting nothing. (Wang)

These findings echo Zhang et al., [31], which emphasized the mediation of learning stress. However, it should be noted that the time constraints may also be attributed to less organized learning. As Wang reported his observation of his classmates:

We generally try to write and submit our essay on the last day of the deadline. If we start to write in the evening, let’s say after eight o’clock or even ten o’clock. It is not possible to be focused because we are rushing to the deadline. So, some used GPT to write an English essay, and then translated into Chinese, and then back into English. I cannot tell who are doing this. (Laughter) (Wang).

Interestingly, students were generally able to differentiate between high-stakes and low-stakes contexts and control the risks of overreliance:

Everybody knows that they can use AI in finishing assignments, but they do not use AI in formal tests. As long as we do not cheat in exams, it is no big deal. (Hu)

AI reduces my thinking and memorization during essay writing. But when I prepare for an important English test, I will abandon AI and try to recite and memorize words. (Zhai)

It is because students have the confidence to control the risks of overreliance, they are willing to continue using AI. However, the confidence in risk control does not necessarily mean actual actions. As Hu commented:

I plan to prepare for the College English Test in this December. So, I plan to stop using AI and study by myself. But if I have no extra time for English learning, I may still rely on AI to write essays. (Hu)

The findings of the study have practical implications for integrating AI into language learning. This study has observed a positive relationship between the perceived risk of overreliance and the intention to continue using GAI. Students compared the overreliance on GAI to “drinking poison to quench thirst”. This metaphor suggests that AI overdependence is inevitable if students have to rush to meet the deadlines of their assignments. Teachers must allow students enough time to complete their learning tasks, and guide them to participate in authentic learning, for example, to keep a reflective journal in the process. The students will be able to compare their own writing and AI output. In addition, formal assessment of L2 writing may need to focus on learning gains, rather than the quality of the finally submitted text. Now that students are generally aware of the potential risks of overreliance on GAI, the key is to provide support to help them minimize the risk of overreliance. Some concrete measures include assigning specific roles and constraints to GAI and asking learners to reflect on their performance, the AI-generated feedback, and their learning gains [33]. More pedagogically sound AI integrations of GAI are desired to serve this purpose [44]. Particular attention should be given to digital literacy education that aims to reduce overreliance on AI and foster adaptive strategies to use AI for personal growth [56].

## Conclusion

This study has explored L2 learners’ risk awareness, a critical ethical concern in the integration of GAI in L2 writing. The study developed a new measure of risk awareness specifically related to L2 writing and established its reliability and validity. The study also explored the associations of risk awareness with students’ attitude toward GAI and their continuance intention. The risk of overreliance negatively predicted attitude but positively predicted continuance intention. The risk of losing teacher feedback positively predicted attitude and indirectly predicted continuance intention. The interviews suggest that the intention to continue using GAI is dependent on the technological affordances of GAI, students’ goal orientations, and the structure of courses and their assignments. This article has important implications for AI integration.

The study is not without limitations. This study included Chinese university students. The results of the study should be interpreted with caution in the Chinese context. The new measure of risk awareness only involved two types of risks that they perceived most relevant. Future research may include participants from a more diverse background and consider other relevant risks. Another limitation is the that the study only considered the intention to continue using GAI for L2 writing. Future research may investigate students’ actual continued usage of GAI and observe the longitudinal changes in usage patterns in response to their developing AI literacy and perceived usefulness.

## Supplementary Information

Supplementary Material 1

## Data Availability

The datasets analyzed during the current study are available from the corresponding author on reasonable request.
