# Supplementary material for: Is it drinking poison to quench thirst? Learners’ risk awareness and continuance intention to use GAI in L2 writing
Source: BMC Psychol. 2026 Mar 12;14:561. doi: 10.1186/s40359-026-04330-x (PMC13094060; doi:10.1186/s40359-026-04330-x)
Supplement: Supplementary file 1 — Supplementary Material 1 [file 40359_2026_4330_MOESM1_ESM.docx]

**Appendix Survey Items**

***Attitude toward AI***

It is a good idea to use AI.

AI makes English writing interesting.

It is fun to interact with AI.

***Continuance Intention***

I will continue to use AI to assist my English writing.

I will continue to learn more about AI so I can skillfully use it.

I plan to continue to use AI tools in the coming days.
